# Systematic, early rhythm control strategy for atrial fibrillation in patients with or without symptoms: the EAST-AFNET 4 trial

**DOI:** 10.1093/eurheartj/ehab593

**Published:** 2021-08-27

**Authors:** Stephan Willems, Katrin Borof, Axel Brandes, Günter Breithardt, A John Camm, Harry J G M Crijns, Lars Eckardt, Nele Gessler, Andreas Goette, Laurent M Haegeli, Hein Heidbuchel, Josef Kautzner, G André Ng, Renate B Schnabel, Anna Suling, Lukasz Szumowski, Sakis Themistoclakis, Panos Vardas, Isabelle C van Gelder, Karl Wegscheider, Paulus Kirchhof

**Affiliations:** Asklepios Hospital St. Georg, Department of Cardiology and Internal intensive care medicine, Faculty of Medicine, Semmelweis University Campus Hamburg, Hamburg, Germany; DZHK (German Center for Cardiovascular Research), Partner Site Hamburg/Kiel/Luebeck, Berlin, Germany; Atrial Fibrillation Network (AFNET), Münster, Germany; Department of Cardiology, University Heart and Vascular Center, University Medical Center Hamburg, Martinistraße 52, Hamburg 20246, Germany; Department of Cardiology, Odense University Hospital, Denmark; Department of Clinical Research, University of Southern Denmark, Odense, Denmark; Atrial Fibrillation Network (AFNET), Münster, Germany; Department of Cardiology II (Electrophysiology), University Hospital Münster, Germany; Cardiology Clinical Academic Group, Molecular and Clinical Sciences Research Institute, St. George’s University of London, UK; Department of Cardiology, Maastricht University Medical Center and Cardiovascular Research Institute Maastricht, Netherlands; Atrial Fibrillation Network (AFNET), Münster, Germany; Department of Cardiology II (Electrophysiology), University Hospital Münster, Germany; Asklepios Hospital St. Georg, Department of Cardiology and Internal intensive care medicine, Faculty of Medicine, Semmelweis University Campus Hamburg, Hamburg, Germany; DZHK (German Center for Cardiovascular Research), Partner Site Hamburg/Kiel/Luebeck, Berlin, Germany; Department of Clinical Research, University of Southern Denmark, Odense, Denmark; St. Vincenz Hospital, Paderborn, Germany; Working Group of Molecular Electrophysiology, University Hospital Magdeburg, Germany; University Hospital Zurich, Zurich, Switzerland; Division of Cardiology, Medical University Department, Kantonsspital Aarau, Switzerland; University Hospital Antwerp and Antwerp University, Antwerp, Belgium; Institute for Clinical and Experimental Medicine, Prague, Czech Republic; Department of Cardiovascular Sciences, University of Leicester, National Institute for Health Research Leicester Biomedical Research Centre, Glenfield Hospital, Leicester, UK; DZHK (German Center for Cardiovascular Research), Partner Site Hamburg/Kiel/Luebeck, Berlin, Germany; Department of Cardiology, University Heart and Vascular Center, University Medical Center Hamburg, Martinistraße 52, Hamburg 20246, Germany; Institute of Medical Biometry and Epidemiology, University Medical Center Hamburg, Eppendorf, Germany; Arrhythmia Center of the National Institute of Cardiology, Medical Division of Cardinal Stefan Wyszynski University in Warsaw, Warsaw, Poland; Department of Cardiology, Ospedale dell’Angelo, Venice, Italy; Heart Sector, Hygeia Hospitals Group, Athens, Greece; University of Groningen, University Medical Center Groningen, Groningen, Netherlands; DZHK (German Center for Cardiovascular Research), Partner Site Hamburg/Kiel/Luebeck, Berlin, Germany; Atrial Fibrillation Network (AFNET), Münster, Germany; Institute for Clinical and Experimental Medicine, Prague, Czech Republic; DZHK (German Center for Cardiovascular Research), Partner Site Hamburg/Kiel/Luebeck, Berlin, Germany; Atrial Fibrillation Network (AFNET), Münster, Germany; Department of Cardiology, University Heart and Vascular Center, University Medical Center Hamburg, Martinistraße 52, Hamburg 20246, Germany; Institute of Cardiovascular Sciences, University of Birmingham, Birmingham, UK

**Keywords:** Atrial fibrillation, Symptoms, Rhythm control, Ablation, Antiarrhythmic drugs, Clinical trial

## Abstract

**Aims:**

Clinical practice guidelines restrict rhythm control therapy to patients with symptomatic atrial fibrillation (AF). The EAST-AFNET 4 trial demonstrated that early, systematic rhythm control improves clinical outcomes compared to symptom-directed rhythm control.

**Methods and results:**

This prespecified EAST-AFNET 4 analysis compared the effect of early rhythm control therapy in asymptomatic patients (EHRA score I) to symptomatic patients. Primary outcome was a composite of death from cardiovascular causes, stroke, or hospitalization with worsening of heart failure or acute coronary syndrome, analyzed in a time-to-event analysis. At baseline, 801/2633 (30.4%) patients were asymptomatic [mean age 71.3 years, 37.5% women, mean CHA_2_DS_2_-VASc score 3.4, 169/801 (21.1%) heart failure]. Asymptomatic patients randomized to early rhythm control (395/801) received similar rhythm control therapies compared to symptomatic patients [e.g. AF ablation at 24 months: 75/395 (19.0%) in asymptomatic; 176/910 (19.3%) symptomatic patients, *P* = 0.672]. Anticoagulation and treatment of concomitant cardiovascular conditions was not different between symptomatic and asymptomatic patients. The primary outcome occurred in 79/395 asymptomatic patients randomized to early rhythm control and in 97/406 patients randomized to usual care (hazard ratio 0.76, 95% confidence interval [0.6; 1.03]), almost identical to symptomatic patients. At 24 months follow-up, change in symptom status was not different between randomized groups (*P* = 0.19).

**Conclusion:**

The clinical benefit of early, systematic rhythm control was not different between asymptomatic and symptomatic patients in EAST-AFNET 4. These results call for a shared decision discussing the benefits of rhythm control therapy in all patients with recently diagnosed AF and concomitant cardiovascular conditions (EAST-AFNET 4; ISRCTN04708680; NCT01288352; EudraCT2010-021258-20).

## Graphical Abstract

**Graphical Abstract:**
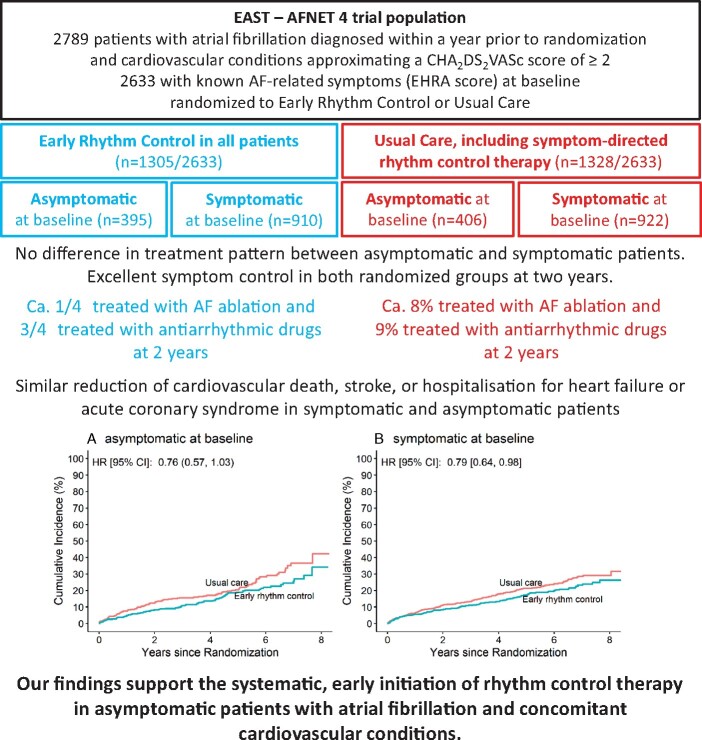

**See the editorial comment for this article ‘Rhythm control in asymptomatic ‘early’ atrial fibrillation: birth of a new paradigm?’, by Robert Hatala, https://doi.org/10.1093/eurheartj/ehab811**.

## Introduction

Atrial fibrillation (AF) remains a major cause of cardiovascular death, stroke, and heart failure even on optimal current management.^1^ Approximately 1/3 of unselected patients with AF do not have AF-related symptoms, more often older populations and with persistent forms of AF.^2^ ^,^ ^3^ Asymptomatic AF is associated with similar rates of stroke, cardiovascular death, and other cardiovascular events compared to symptomatic AF.^4–7^ Some data suggest that the mortality of asymptomatic patients may even be higher than in symptomatic patients,^4^ underscoring the need to better identify and manage asymptomatic patients with AF.

Contemporary AF guidelines recommend anticoagulation and therapy of concomitant cardiovascular conditions in all patients with AF, while rhythm control is restricted to symptomatic patients.^1^ However, the EAST-AFNET 4 trial and the earlier ATHENA trial suggest that rhythm control therapy could further reduce cardiovascular events, reporting fewer cardiovascular complications in patients randomized to early rhythm control therapy (EAST-AFNET 4)^8^ or to dronedarone (ATHENA).^9^ Whether the clinical benefit of systematic, early rhythm control therapy is present in asymptomatic patients with AF remains to be tested.

## Methods

The current analysis was prespecified in the statistical analysis plan and performed on the final, locked database of the EAST-AFNET 4 trial. Design and topline results of the main trial have been published.^8^ ^,^ ^10^ In brief, the EAST-AFNET 4 trial is an international, investigator-initiated, parallel-group, open, blinded- outcome-assessment (PROBE) trial, which randomly assigned patients who had AF diagnosed ≤1 year before enrolment and cardiovascular conditions to receive either early rhythm control in all patients or usual care. Early rhythm control included treatment with antiarrhythmic drugs or AF ablation in all patients directly after randomization. Usual care included rhythm control therapy to improve AF-related symptoms.^8^ ^,^ ^10^

The current analyses included treatments at discharge from the randomization visit, at one year and at two years of follow-up. Patients were categorized into asymptomatic and symptomatic patients by EHRA score at baseline (asymptomatic = EHRA I; symptomatic = EHRA II–IV). To further explore relations between symptoms and outcomes, patients were classified as asymptomatic (EHRA I), mildly symptomatic (EHRA II), or severely symptomatic (EHRA III or EHRA IV). Patients with missing baseline EHRA scores were excluded.

The effects of early rhythm control therapy were compared between randomized groups (ITT analysis) in patients with different EHRA classes and between asymptomatic and symptomatic patients at baseline. Effects on the primary outcome (composite of death from cardiovascular causes, stroke, or hospitalization with worsening of heart failure or acute coronary syndrome, analyzed in a time-to-event analysis) as well as the second primary outcome (number of nights spent in hospital per year) and key secondary outcomes (rhythm, change in symptoms, left ventricular function, quality of life) were analyzed.

Data are presented as mean and standard deviation or number and percentage. To compare data, *P*-values resulting from mixed linear regression models for metric variables and mixed (ordinal) logistic regression models for categorical variables were used where appropriate. Sites were modelled as random effect. For categorical variables with more than two categories (not ordinal), a random effect was not included. Subgroup analyses were conducted with interaction terms.

For the primary outcome and its individual components, we used Cox regression models with an interaction term of treatment group and AF symptom status as well as site as a shared frailty term. The treatment effects are expressed as hazard ratios with 95% confidence interval.

The second primary outcome was analyzed with a mixed negative binomial model with treatment group and AF symptom status as interaction term, the log of follow-up time as offset and site as a random effect. The treatment effect is presented as the incidence rate ratio and 95% confidence interval. The key secondary outcomes at 2 years were analyzed after multiple imputation of missing values in survivors. The multiple-imputation procedure was conducted with 60 imputations for a set of continuous outcomes and covariates for adjustment based on suggestions by White, Royston, and Wood (see statistical analysis plan in the supplement of EAST-AFNET 4).^8^ We then calculated a mixed linear model with the corresponding baseline measurement as covariate, a treatment group/AF symptom interaction term and site as a random effect. The treatment effect is expressed as the adjusted mean difference with 95% confidence interval. Data were analyzed using Stata software (StataCorp), version 16.1, and R software, version 4.0.5 (R Project for Statistical Computing).

## Results

**Figure 1:**
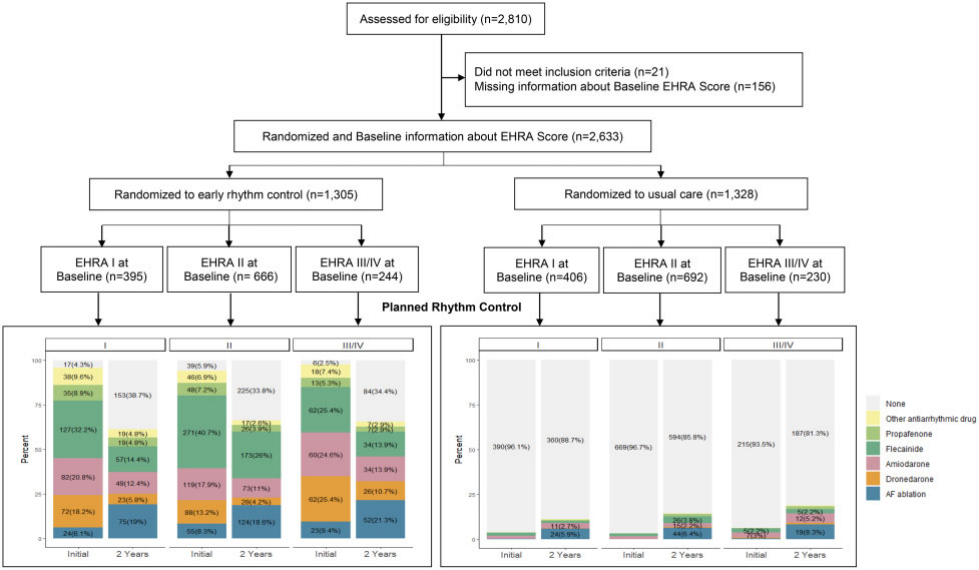
Consort flow chart of the patients included in this analysis showing screening, randomization, treatment, and follow-up. Of all patients included into the EAST-AFNET 4 trial, 21 of the 2810 patients did not meet the inclusion criteria of early atrial fibrillation (diagnosed ≤1 year before enrolment) and cardiovascular conditions. After exclusion of 156 patients with missing baseline symptom status, 2633 patients were included into the analysis with randomization to early rhythm control (*n* = 1305) or usual care (*n* = 1328). Most of the patients assigned to early rhythm-control were initially treated with antiarrhythmic drugs, regardless of symptom status. After 2 years of follow-up, 242 of the 395 asymptomatic patients (59.3%) and 601 of the 910 symptomatic patients (64.8%) who had been randomly assigned to early rhythm control therapy were still receiving active rhythm-control therapy [atrial fibrillation ablation in 75/395 (19.0%) asymptomatic patients and in 176/910 (19.3%) symptomatic patients; *P* = 0.672] randomized to early rhythm control. This corresponds to ca 25% of patients randomized to early rhythm control and still in follow-up at 24 months.

The study group consisted of 2633 patients randomized across 135 sites in 11 European countries between 28 July 2011 and 30 December 2016. Patients with missing baseline symptom status (156, 2.8%) were not analyzed (*Figure 1*). At baseline, 801/2633 (30.4%) patients were asymptomatic (EHRA score I, mean age 71.3 years, 37.5% women, mean CHA_2_DS_2_-VASc score 3.4), whereas 1832/2633 (69.6%) patients were symptomatic (*Table 1* and Supplementary material online, *Tables S1 and S2*). The majority of symptomatic patients presented with mild or moderate symptoms (EHRA II, n = 1358, *Table 1*). The mean CHA_2_DS_2_-VASc score was not different between symptomatic and asymptomatic patients. However, asymptomatic patients were older, less often women, had less heart failure but had higher rates of prior stroke or transient ischaemic attack compared to symptomatic patients (*Table 1*). AF patterns and prior rhythm control therapy also differed. Asymptomatic patients were more often enrolled at their first episode of AF and were less likely to be in paroxysmal AF (*Table 1*). Asymptomatic patients had fewer rhythm control therapy attempts prior to enrolment using antiarrhythmic drugs or electrical or pharmacological cardioversion. Baseline characteristics of asymptomatic patients were well balanced between randomized groups (*Table 1*).

**Table 1:** Demographic and clinical characteristics of the patients at baseline

Characteristics	Overall, *N* = 2633^a^	Symptom status at baseline (EHRA Class)			*P*-value^b^
		Asymptomatic (EHRA I), *N* = 801^a^	Mild or moderate symptoms (EHRA II), *N* = 1358^a^	Severe symptoms (EHRA III/IV), *N* = 474^a^	
Age	71 (66.0, 76)	72 (67.0, 77)	71 (65.2, 75)	72 (65.0, 76)	0.003
Female sex	1223/2633 (46%)	300/801 (37%)	629/1358 (46%)	294/474 (62%)	<0.001
Body mass index (calculated) (kg/m^2^)	28.6 (25.5, 32.1)	28.4 (25.6, 32.0)	28.3 (25.4, 31.8)	29.4 (25.8, 33.1)	0.047
AF type					<0.001
First episode	1029/2633 (39%)	390/801 (49%)	442/1358 (33%)	197/474 (42%)	
Paroxysmal	901/2633 (34%)	201/801 (25%)	532/1358 (39%)	168/474 (35%)	
Persistent or long-standing persistent	703/2633 (27%)	210/801 (26%)	384/1358 (28%)	109/474 (23%)	
Sinus rhythm at baseline	1424/2632 (54%)	401/800 (50%)	742/1358 (55%)	281/474 (59%)	0.079
Median days since AF diagnosis (IQR)	35.0 (6.0, 110.0)	23.0 (4.0, 86.0)	44.0 (8.0, 124.8)	24.0 (4.0, 103.8)	0.002
Previous pharmacological or electrical cardioversion	1046/2602 (40%)	276/788 (35%)	564/1346 (42%)	206/468 (44%)	<0.001
Concomitant cardiovascular conditions					
Previous stroke or transient ischaemic attack	303/2633 (12%)	116/801 (14%)	140/1358 (10%)	47/474 (9.9%)	0.014
At least mild cognitive impairment	1110/2524 (44%)	383/777 (49%)	514/1309 (39%)	213/438 (49%)	0.007
MoCA score					<0.001
None	1414/2524 (56%)	394/777 (51%)	795/1309 (61%)	225/438 (51%)	
Mild	1016/2524 (40%)	343/777 (44%)	482/1309 (37%)	191/438 (44%)	
Moderate	86/2524 (3.4%)	38/777 (4.9%)	29/1309 (2.2%)	19/438 (4.3%)	
Severe	8/2524 (0.3%)	2/777 (0.3%)	3/1309 (0.2%)	3/438 (0.7%)	
Arterial hypertension	2306/2633 (88%)	693/801 (87%)	1194/1358 (88%)	419/474 (88%)	0.64
Systolic blood pressure (mmHg)	135 (123.0, 150)	137 (123.0, 150)	135 (123.0, 149)	137 (122.0, 150)	0.23
Diastolic blood pressure (mmHg)	80 (73.0, 90)	80 (72.0, 90)	80 (74.0, 90)	80 (70.0, 89)	0.023
Stable heart failure	738/2633 (28%)	169/801 (21%)	396/1358 (29%)	173/474 (36%)	<0.001
CHA_2_DS_2_-Vasc score	3.0 (2.0, 4.0)	3.0 (2.0, 4.0)	3.0 (2.0, 4.0)	3.0 (3.0, 4.0)	<0.001
Chronic kidney disease of MDRD stage 3 or 4	337/2633 (13%)	104/801 (13%)	171/1358 (13%)	62/474 (13%)	0.72
Medication at discharge					
Oral anticoagulation with NOAC or VKA	2378/2633 (90%)	723/801 (90%)	1223/1358 (90%)	432/474 (91%)	0.11
Digoxin or digitoxin	129/2633 (4.9%)	24/801 (3.0%)	77/1358 (5.7%)	28/474 (5.9%)	0.003
Beta-blockers	2130/2633 (81%)	624/801 (78%)	1099/1358 (81%)	407/474 (86%)	0.005
ACE inhibitors or angiotensin II receptor blocker	1838/2633 (70%)	580/801 (72%)	929/1358 (68%)	329/474 (69%)	0.41
Mineralocorticoid receptor antagonist	170/2633 (6.5%)	53/801 (6.6%)	88/1358 (6.5%)	29/474 (6.1%)	0.74
Diuretic	1067/2633 (41%)	322/801 (40%)	521/1358 (38%)	224/474 (47%)	0.004
Statin	1139/2633 (43%)	399/801 (50%)	556/1358 (41%)	184/474 (39%)	0.002
Platelet inhibitor	437/2633 (17%)	146/801 (18%)	210/1358 (15%)	81/474 (17%)	0.24
Planned therapy for rhythm control at baseline					0.15
AAD	1193/2633 (45%)	369/801 (46%)	595/1358 (44%)	229/474 (48%)	
Ablation	104/2633 (3.9%)	25/801 (3.1%)	55/1358 (4.1%)	24/474 (5.1%)	
None	1336/2633 (51%)	407/801 (51%)	708/1358 (52%)	221/474 (47%)	

MoCA score categories: none: ≥26; mild: 18–25; moderate: 10–17; severe: <10.

AF, atrial fibrillation.

a Median (IQR) or frequency with number/total number (%).

b *P*-values resulting from mixed linear or logistic regression models and Analysis of Deviance Table (Type II Wald chi-square tests). Nominal variables were tested with Pearson's Chi-squared test. AAD, antiarrhythmic drug; BL, baseline; CI, confidence interval; IQR, interquartile range; HF, heart failure; ITT, intention to treat; MDRD, Modification of Diet in Renal Disease; MoCA, Montréal Cognitive Assessment; NOAC, non vitamin-K-antagonist oral anticoagulant; VKA, vitamin K antagonist.

### Anticoagulation and treatment of concomitant cardiovascular conditions

Most asymptomatic patients received guideline-adherent oral anticoagulation, comparable to the care in the symptomatic group at baseline [baseline: asymptomatic 723/801 (90.3%), symptomatic 1655/1832 (90.3%)] and during follow-up (Supplementary material online, *Table S2*). Similar to symptomatic patients, treatment of concomitant cardiovascular conditions was well balanced between groups with the exception of slightly lower use of beta-blockers and digitalis glycosides and higher use of statins in asymptomatic patients (*Table 1* and Supplementary material online, *Table S2*, *P* = 0.004, *P* = 0.001, and *P* < 0.001, respectively).

### Delivery of early rhythm control and usual care in asymptomatic patients

Of 801 asymptomatic patients, 395 were assigned to early rhythm control and 406 to usual care (*Figure 1*). Rhythm control therapy was initiated in nearly all asymptomatic patients [378/395 (95.7%)] randomized to early rhythm control without difference to symptomatic patients [865/910 (95.1%)] and without effect of symptom status. At the two year follow-up, 242/395 (59.3%) asymptomatic patients were still receiving rhythm control therapy, compared to 601/910 (64.8%) symptomatic patients (*Figure 1* and *Table 2*). Asymptomatic patients randomized to early rhythm control (395/801) received similar rhythm control therapies compared to symptomatic patients. A total of 75/395 (19.0%) asymptomatic patients received AF ablation within 24 months after randomization compared to 176/910 (19.3%) symptomatic patients (*P* = 0.672, *Table 2*). This corresponds to 25% of patients still in follow-up at two years.

**Table 2:** Rhythm control planned at baseline and present at 24 months by EHRA score and randomized

	EHRA I		EHRA II		EHRA III/IV	
	Early rhythm control	Usual care	Early rhythm control	Usual care	Early rhythm control	Usual care
*n*	395	406	666	692	244	230
Rhythm control at BL						
AF ablation	24 (6.1)	1 (0.2)	55 (8.3)	0 (0.0)	23 (9.4)	1 (0.4)
Dronedarone	72 (18.2)	0 (0.0)	88 (13.2)	0 (0.0)	62 (25.4)	1 (0.4)
Amiodarone	82 (20.8)	7 (1.7)	119 (17.9)	12 (1.7)	60 (24.6)	7 (3.0)
Flecainide	127 (32.2)	6 (1.5)	271 (40.7)	10 (1.4)	62 (25.4)	5 (2.2)
Propafenone	35 (8.9)	1 (0.2)	48 (7.2)	1 (0.1)	13 (5.3)	0 (0.0)
Other antiarrhythmic drug	38 (9.6)	1 (0.2)	46 (6.9)	0 (0.0)	18 (7.4)	1 (0.4)
None	17 (4.3)	390 (96.1)	39 (5.9)	669 (96.7)	6 (2.5)	215 (93.5)
Rhythm control at FU24						
AF ablation	75 (19.0)	24 (5.9)	124 (18.6)	44 (6.4)	52 (21.3)	19 (8.3)
Dronedarone	23 (5.8)	1 (0.2)	28 (4.2)	2 (0.3)	26 (10.7)	2 (0.9)
Amiodarone	49 (12.4)	11 (2.7)	73 (11.0)	15 (2.2)	34 (13.9)	12 (5.2)
Flecainide	57 (14.4)	5 (1.2)	173 (26.0)	26 (3.8)	34 (13.9)	5 (2.2)
Propafenone	19 (4.8)	3 (0.7)	26 (3.9)	9 (1.3)	7 (2.9)	4 (1.7)
Other antiarrhythmic drug	19 (4.8)	2 (0.5)	17 (2.6)	2 (0.3)	7 (2.9)	1 (0.4)
None	153 (38.7)	360 (88.7)	225 (33.8)	594 (85.8)	84 (34.4)	187 (81.3)

AF, atrial fibrillation; FU24, 24 months follow-up.

Treatment patterns in patients randomized to usual care did not differ between symptomatic and asymptomatic patients. Rate control therapy without rhythm control therapy was given to 390 (96.1%) asymptomatic patients randomized to usual care and to 884/922 (95.9%) symptomatic patients. At 2 years, 360/406 patients (89.3%) randomized to usual care were still not receiving rhythm-control therapy, slightly more than in symptomatic patients randomized to usual care [781/922 (85.9%), *P* = 0.0487, *Figure 1* and *Table 2*].

### Primary outcome is not different based on symptoms

**Figure 2:**
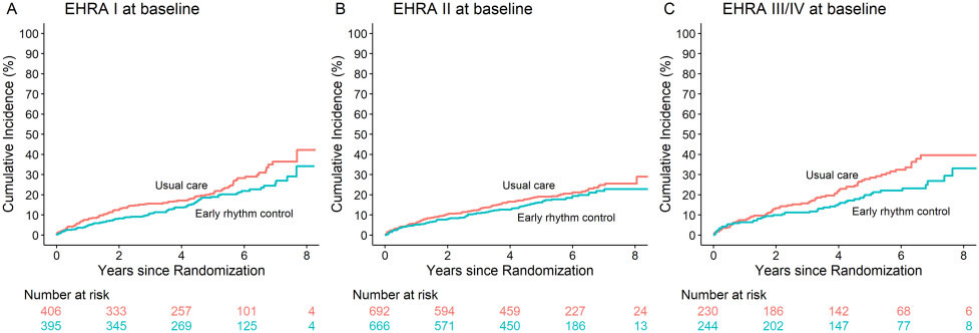
Aalen–Johansen cumulative-incidence curves for the first primary outcome: (*A*) for patients with asymptomatic atrial fibrillation at baseline and (*B*) for patients with symptomatic atrial fibrillation at baseline. The first primary outcome was a composite of death from cardiovascular causes, stroke, or hospitalization with worsening of heart failure or acute coronary syndrome. The effect of early rhythm control on this outcome was almost identical in symptomatic and asymptomatic patients.

Death from cardiovascular causes, stroke, or hospitalization with worsening of heart failure or acute coronary syndrome (first primary outcome of the trial) occurred in 79/395 asymptomatic patients randomized to early rhythm control, and in 97/406 asymptomatic patients randomized to usual care, with a hazard ratio of 0.76, 95% CI [0.57; 1.03], almost identical to the overall population and to symptomatic patients (*P* = 0.848, *Figure 2* and *Table 3*) and to the outcome in patients with mild to moderate or severe symptoms (*Figure 2* and *Table 3*). The effects of early rhythm control on individual components of the first primary outcome were comparable for asymptomatic, mildly or moderately symptomatic, and severely symptomatic patients, in line with the overall population (*Table 3*). There was no interaction between symptom status and treatment effect in the primary outcome or any of its components.

**Table 3:** Outcomes in the EAST – AFNET 4 trial population by symptom status

Outcome	Asymptomatic (EHRA I)			Mild or moderate symptoms (EHRA II)			Severe symptoms (EHRA III/IV)			*P*-value interaction
	Early rhythm control	Usual care	Treatment effect	Early rhythm control	Usual care	Treatment effect	Early rhythm control	Usual care	Treatment effect	
First primary outcome	79/1888 (4.2)	97/1774 (5.5)	0.77 (0.57, 1.03)	109/3056 (3.6)	97/1774 (5.5)	0.84 (0.66, 1.09)	49/1099 (4.5)	68/1031 (6.6)	0.68 (0.47, 0.99)	0.743
Components of the first primary outcome										
Death from cardiovascular causes	19/2049 (0.9)	22/1977 (1.1)	0.82 (0.44, 1.52)	30/3277 (0.9)	22/1977 (1.1)	0.73 (0.46, 1.17)	15/1208 (1.2)	22/1189 (1.9)	0.68 (0.35, 1.31)	0.973
Stroke	19/2005 (0.9)	25/1923 (1.3)	0.73 (0.4, 1.33)	14/3242 (0.4)	25/1923 (1.3)	0.7 (0.36, 1.37)	6/1188 (0.5)	12/1151 (1)	0.48 (0.18, 1.27)	0.663
Hospitalization with worsening of heart failure	39/1961 (2)	54/1845 (2.9)	0.67 (0.44, 1.01)	62/3148 (2)	54/1845 (2.9)	0.96 (0.68, 1.35)	31/1141 (2.7)	39/1082 (3.6)	0.76 (0.47, 1.22)	0.432
Hospitalization with acute coronary syndrome	22/2004 (1.1)	18/1929 (0.9)	1.19 (0.64, 2.22)	18/3208 (0.6)	18/1929 (0.9)	0.61 (0.34, 1.09)	10/1180 (0.8)	12/1148 (1)	0.82 (0.35, 1.89)	0.229
Secondary primary outcome—nights spent in hospital/yr	5.5 (17.9)	6.1 (19.2)	0.91 (0.72, 1.16)	5.3 (20.7)	6.1 (19.2)	1.19 (0.99, 1.43)	8.9 (32.4)	5.8 (13.8)	1.19 (0.87, 1.62)	0.193
Key secondary outcomes at 2 years										
Change in left ventricular ejection fraction	0.6 (10.4)	−0.5 (9.8)	0.18 (−1, 1.36)	1.4 (9.4)	−0.5 (9.8)	0.14 (−0.82, 1.1)	3.5 (10.6)	0.9 (11.7)	0.56 (−1.06, 2.17)	0.902
Change in EQ-5D score	1.6 (16.7)	−1.2 (17.2)	1.53 (−1.74, 4.8)	1.1 (16.8)	−1.2 (17.2)	1.31 (−1.26, 3.89)	1.6 (19.6)	4 (19.1)	−0.25 (−4.58, 4.08)	0.797
Change in SF-12 Mental Score	1 (9.7)	1.7 (9.5)	−0.83 (−2.33, 0.68)	0.1 (10.3)	1.7 (9.5)	−1.37 (−2.5, −0.24)	2.6 (12.2)	2.8 (10.9)	−1.3 (−3.3, 0.71)	0.848
Change in SF-12 Physical Score	−0.4 (8.3)	−1.2 (8.4)	0.84 (−0.57, 2.24)	0.5 (8.3)	−1.2 (8.4)	0.02 (−0.96, 1.01)	1.3 (9.1)	1.3 (8.9)	0.39 (−1.3, 2.08)	0.636
Change in MoCA score	0.2 (3.4)	0.1 (3)	0.05 (−0.42, 0.53)	0.1 (3.2)	0.1 (3)	−0.06 (−0.42, 0.3)	−0.1 (3.4)	0.1 (3.1)	−0.66 (−1.31, 0)	0.194
Sinus rhythm	255/323 (78.9)	170/325 (52.3)	3.65 (2.56, 5.22)	450/538 (83.6)	170/325 (52.3)	3.21 (2.41, 4.28)	166/191 (86.9)	141/185 (76.2)	2.12 (1.2, 3.75)	0.287

Safety outcomes for asymptomatic patients were similar to symptomatic patients with no significant difference between groups (*Table 4*). Mortality and stroke were similar in the two treatment groups. Serious adverse events related to rhythm-control therapy were rare, more common in the group assigned to early rhythm control, and not affected by symptom status.

**Table 4:** Safety outcomes for EHRA score

	EHRA I		EHRA II		EHRA III/IV	
	Early rhythm control	Usual care	Early rhythm control	Usual care	Early rhythm control	Usual care
*n*	395	406	666	692	244	230
Primary composite safety outcome	79 (20.0)	63 (15.5)	99 (14.9)	105 (15.2)	44 (18.0)	43 (18.7)
Stroke	19 (4.8)	25 (6.2)	14 (2.1)	22 (3.2)	6 (2.5)	12 (5.2)
Death	45 (11.4)	40 (9.9)	59 (8.9)	84 (12.1)	29 (11.9)	30 (13.0)
Serious adverse event of special interest related to rhythm control therapy	22 (5.6)	4 (1.0)	31 (4.7)	8 (1.2)	12 (4.9)	5 (2.2)
Serious adverse event related to antiarrhythmic drug therapy						
Nonfatal cardiac arrest	0 (0.0)	0 (0.0)	1 (0.2)	0 (0.0)	0 (0.0)	1 (0.4)
Drug induced bradycardia	0 (0.0)	0 (0.0)	7 (1.1)	3 (0.4)	5 (2.0)	1 (0.4)
Torsade de pointes tachycardia	0 (0.0)	0 (0.0)	1 (0.2)	0 (0.0)	0 (0.0)	0 (0.0)
Drug toxicity of AF-related drug therapy	3 (0.8)	2 (0.5)	5 (0.8)	0 (0.0)	2 (0.8)	1 (0.4)
Atrioventricular block	1 (0.3)	0 (0.0)	1 (0.2)	0 (0.0)	0 (0.0)	0 (0.0)
Serious adverse event related to AF ablation						
Pericardial tamponade	0 (0.0)	0 (0.0)	2 (0.3)	0 (0.0)	1 (0.4)	0 (0.0)
Blood pressure related event	1 (0.3)	0 (0.0)	0 (0.0)	0 (0.0)	0 (0.0)	0 (0.0)
Syncope	1 (0.3)	0 (0.0)	2 (0.3)	0 (0.0)	1 (0.4)	1 (0.4)
Other serious adverse event of special interest related to rhythm control therapy						
Other event	1 (0.3)	0 (0.0)	0 (0.0)	0 (0.0)	0 (0.0)	2 (0.9)
Other cardiovascular event	4 (1.0)	0 (0.0)	1 (0.2)	0 (0.0)	0 (0.0)	0 (0.0)
Major bleeding related to AF ablation	3 (0.8)	0 (0.0)	3 (0.5)	0 (0.0)	0 (0.0)	0 (0.0)
Hospitalization for AF	4 (1.0)	0 (0.0)	6 (0.9)	3 (0.4)	1 (0.4)	0 (0.0)
Nonmajor bleeding related to AF ablation	0 (0.0)	1 (0.2)	1 (0.2)	1 (0.1)	0 (0.0)	0 (0.0)
Hospitalization for worsening of HF with decompensated HF	2 (0.5)	0 (0.0)	1 (0.2)	0 (0.0)	0 (0.0)	0 (0.0)
Implantation of a pacemaker, defibrillator or other	3 (0.8)	1 (0.2)	1 (0.2)	2 (0.3)	3 (1.2)	1 (0.4)

AF, atrial fibrillation.

### Effect of early rhythm control therapy on nights spent in hospital and on secondary outcomes in asymptomatic and symptomatic patients

There was no difference in nights spent in hospital per year between asymptomatic, mildly or moderately symptomatic, and severely symptomatic patients (*Table 3*). Furthermore, nights spent in hospital per year were not different between asymptomatic patients randomized to early rhythm control (5.5 ± 17.9) compared to asymptomatic patients randomized to usual care (6.1 ± 19.2). At 24 months, 255/323 (78.9%) asymptomatic patients in early rhythm control group were in sinus rhythm compared to 170/325 (52.3%) patients in usual care (*P* < 0.001), similar to the results of symptomatic patients (*Table 3*). There was no interaction between symptom status and treatment effect in any of the secondary outcomes (*Table 3*).

**Figure 3:**
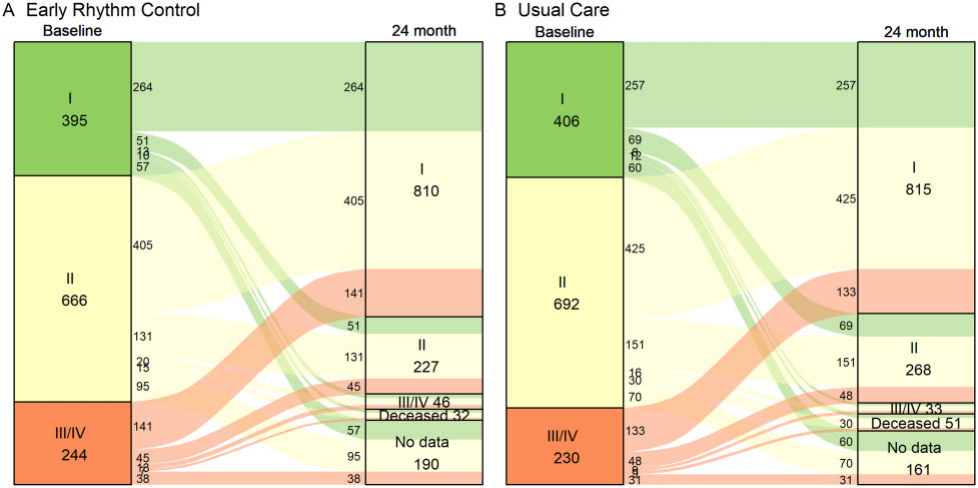
Change in atrial fibrillation symptoms between discharge from baseline and 24 months follow-up. There were no differences in symptom status at 24 months between randomized groups, irrespective of the presence of baseline symptoms (*P* = 0.1161). Symptoms improved without inter-group difference, illustrating the good adherence to protocol in patients randomized to usual care, which included symptom-directed rhythm control therapy to improve atrial fibrillation-related symptoms.

Most patients who were asymptomatic at baseline remained asymptomatic. Only 144/687 (21%) of initially asymptomatic patients became symptomatic in the first 2 years of follow-up, with no difference between randomized groups (*P* = 0.19, *Figure 3*). In symptomatic patients, AF symptoms decreased over time with 1104/1832 (60.3%) initially symptomatic patients becoming asymptomatic during follow-up.

## Discussion

### Main findings

This prespecified comparison of the effect of early rhythm control therapy in asymptomatic patients with AF randomized in the EAST-AFNET 4 trial yielded several new findings:

1. Asymptomatic and symptomatic patients share many clinical characteristics, including similar CHA_2_DS_2_VASc scores with slight differences in the type of comorbidities and more first diagnosed or persistent forms of AF in asymptomatic patients.
2. Anticoagulation and treatment of concomitant cardiovascular conditions was not different between asymptomatic and symptomatic patients.
3. Asymptomatic patients randomized in EAST-AFNET 4 received an almost identical early rhythm control therapy compared to symptomatic patients, including AF ablation in approximately a quarter of patients still in follow-up at 2 years after randomization.
4. The effect of rhythm control therapy on cardiovascular complications in asymptomatic patients with AF is not different to the effect in symptomatic patients and is not affected by symptom severity.

Our findings support systematic, early initiation of rhythm control therapy in all patients with AF and concomitant cardiovascular conditions independent of their AF-related symptoms (*Graphical Abstract*).

Current treatment guidelines recommend rhythm control therapy in symptomatic patients with AF only,^1^ ^,^ ^11^ excluding most asymptomatic patients from this therapy.^7^ These recommendations are based on earlier ‘rate vs. rhythm’ trials showing no effect of rhythm control therapy in cardiovascular outcomes,^12–14^ including a neutral outcome in patients with recently diagnosed AF randomized to rhythm control therapy in AFFIRM.^15^ Later, the ATHENA trial randomized asymptomatic and symptomatic patients with AF to treatment with dronedarone or placebo and found a reduction in a composite of death and cardiovascular hospitalization, providing a first signal that rhythm control therapy could convey clinical benefit in patients with AF.^9^ This signal was also detected in the CASTLE-AF trial comparing AF ablation to medical therapy in patients with AF and severe heart failure.^16^ Both trials mainly enrolled symptomatic patients and, unlike AFFIRM, enrolled patients on continued anticoagulation. On the other hand, recent data demonstrate the safety of modern rhythm control therapy, including catheter ablation.^17–21^ However, these results were obtained in symptomatic patients with a current indication for rhythm control therapy. The main findings of ATHENA,^9^ CASTLE-AF,^16^ and EAST-AFNET 4^8^ suggest a potential clinical benefit of early rhythm control therapy, providing a sound rationale to use rhythm control therapy in asymptomatic patients with AF. The present analysis demonstrates that early and systematic initiation of rhythm control therapy in asymptomatic patients with AF enrolled in a variety of clinical settings conveys the same clinical benefit as in symptomatic patients. Further details of the therapies used to deliver the early rhythm control treatment strategy, which included AF ablation in 25% of patients randomized to early rhythm control and still in follow-up at two years, and antiarrhythmic drugs in 75% of patients, have been published.^22^

### Clinical characteristics of asymptomatic patients with AF

Asymptomatic patients enrolled into EAST-AFNET 4 were older than symptomatic patients, had a similar CHA_2_DS_2_VASc score, but with slight differences in risk factor profiles, and were less frequently in paroxysmal AF (*Table 1*). This is different from the asymptomatic patients enrolled into AFFIRM and RACE who tended to be younger and had less comorbidities than symptomatic patients in those trials.^23^ ^,^ ^24^ The clinical characteristics of the asymptomatic AF population in EAST-AFNET 4 replicate features of asymptomatic patients in contemporary European and American general AF registries.^4–7^ These similarities suggest that the findings of this subanalysis are applicable to general patients with asymptomatic AF.

Asymptomatic patients had fewer attempts of rhythm control therapy at the time of enrolment into EAST-AFNET 4, which is in line with the current guideline recommendations restricting rhythm control therapy to the improvement of symptoms in symptomatic patients with AF.^1^ ^,^ ^11^ This observation underscores the potential impact of the present analysis demonstrating a relevant reduction in the composite of cardiovascular death, stroke, or hospitalization for heart failure or acute coronary syndrome in asymptomatic patients with AF. Almost all patients enrolled into EAST-AFNET 4 had clinically diagnosed AF, which could include AF detected during routine medical visits, e.g. during vaccination. These results call for further research into the value of rhythm control therapy in patients undergoing active screening for AF.

### Symptoms and quality of life in asymptomatic atrial fibrillation

The positive effect of early rhythm control on the primary outcome was not associated with improved quality of life in EAST-AFNET 4, neither in symptomatic nor in asymptomatic patients. While details of this observation remain to be analyzed, a plausible explanation is the comparator (‘usual care’) in EAST-AFNET 4: Patients randomized to usual care received rhythm control therapy to improve symptoms on optimal rate control.^10^ About 15% of patients randomized to usual care received rhythm control therapy. This symptom-directed and delayed rhythm control therapy in patients randomized to usual care can explain the high proportion of patients with well-controlled symptoms in both randomized groups in the trial. It is possible that a higher use of AF ablation, used in 25% of asymptomatic patients with AF who were randomized to early therapy and still in follow-up at two years in this analysis, and in a similar proportion in the overall trial, could have improved quality of life even further.^19^ ^,^ ^25–27^ This should be studied in future trials.

Most patients who were included in EAST-AFNET 4 without AF-related symptoms remained asymptomatic. Only 21.7% of initially asymptomatic patients became symptomatic during follow-up, with no difference between treatment groups. The results of this analysis demonstrate that systematic, early rhythm control therapy reduces cardiovascular outcomes in patients with recently diagnosed AF irrespective of AF-related symptoms. These facts invite a fundamental rethink regarding the treatment of AF. In our view, these findings call for the inclusion of rhythm control therapy early and systematically in asymptomatic patients with AF and concomitant cardiovascular conditions.

### Limitations

Although the EAST-AFNET 4 trial was a randomized, multicentre controlled trial, it was not powered for a primary analysis of asymptomatic patients. The directionality and the magnitude of the effect on the primary outcome and of its components were comparable to the effect in symptomatic patients and in the overall trial population. The 95% confidence interval of the hazard ratio for the primary outcome (0.76 [0.57, 1.03]) included 1, as could be expected due to the lower number of patients in this subgroup. Symptoms can be difficult to assess, and asymptomatic patients occasionally report improved symptoms after restoration of sinus rhythm. Furthermore, symptom assessment in this subanalysis was limited to the EHRA score, a validated^26^ but general instrument capturing symptoms related to AF.

## Conclusions

These results call for a shared decision process discussing the benefits of rhythm control therapy in all patients with recently diagnosed AF and concomitant cardiovascular conditions regardless of their AF-related symptoms (EAST-AFNET 4 ISRCTN number, ISRCTN04708680; ClinicalTrials.gov number, NCT01288352; EudraCT number, 2010-021258-20).

## Supplementary material

Supplementary material is available at *European Heart Journal* online.

## Supplementary Material

ehab593_Supplementary_Data

## References

[1] Hindricks G, Potpara T, Dagres N, Arbelo E, Bax JJ, Blomstrom-Lundqvist C, Boriani G, Castella M, Dan GA, Dilaveris PE, Fauchier L, Filippatos G, Kalman JM, La Meir M, Lane DA, Lebeau JP, Lettino M, Lip GYH, Pinto FJ, Thomas GN, Valgimigli M, Van Gelder IC, Van Putte BP, Watkins CL; ESCSD Group. 2020 ESC Guidelines for the diagnosis and management of atrial fibrillation developed in collaboration with the European Association for Cardio-Thoracic Surgery (EACTS). Eur Heart J 2021;42:373–498. 32860505 10.1093/eurheartj/ehaa612

[2] Xiong Q, Proietti M, Senoo K, Lip GY. Asymptomatic versus symptomatic atrial fibrillation: a systematic review of age/gender differences and cardiovascular outcomes. Int J Cardiol 2015;191:172–177. 25974193 10.1016/j.ijcard.2015.05.011

[3] Schnabel RB, Pecen L, Ojeda FM, Lucerna M, Rzayeva N, Blankenberg S, Darius H, Kotecha D, Caterina R, Kirchhof P. Gender differences in clinical presentation and 1-year outcomes in atrial fibrillation. Heart 2017;103:1024–1030. 28228467 10.1136/heartjnl-2016-310406 PMC5529986

[4] Boriani G, Laroche C, Diemberger I, Fantecchi E, Popescu MI, Rasmussen LH, Sinagra G, Petrescu L, Tavazzi L, Maggioni AP, Lip GY. Asymptomatic atrial fibrillation: clinical correlates, management, and outcomes in the EORP-AF Pilot General Registry. Am J Med 2015;128:509–18. e2. 25534423 10.1016/j.amjmed.2014.11.026

[5] Thind M, Holmes DN, Badri M, Pieper KS, Singh A, Blanco RG, Steinberg BA, Fonarow GC, Gersh BJ, Mahaffey KW, Peterson ED, Reiffel JA, Piccini JP, Kowey PR; ORBIT-AF Investigators and Patients. Embolic and other adverse outcomes in symptomatic versus asymptomatic patients with atrial fibrillation (from the ORBIT-AF Registry). Am J Cardiol 2018;122:1677–1683. 30227964 10.1016/j.amjcard.2018.07.045

[6] Gibbs H, Freedman B, Rosenqvist M, Virdone S, Mahmeed WA, Ambrosio G, Camm AJ, Jacobson B, Jerjes-Sanchez C, Kayani G, Oto A, Panchenko E, Ragy H, Kakkar AK; GARFIELD-AF Investigators. Clinical outcomes in asymptomatic and symptomatic atrial fibrillation presentations in GARFIELD-AF: implications for AF screening. Am J Med 2021;134:893–901. 33607088 10.1016/j.amjmed.2021.01.017

[7] Arnar DO, Mairesse GH, Boriani G, Calkins H, Chin A, Coats A, Deharo JC, Svendsen JH, Heidbuchel H, Isa R, Kalman JM, Lane DA, Louw R, Lip GYH, Maury P, Potpara T, Sacher F, Sanders P, Varma N, Fauchier L; ESCSD Group; ESD Committee. Management of asymptomatic arrhythmias: a European Heart Rhythm Association (EHRA) consensus document, endorsed by the Heart Failure Association (HFA), Heart Rhythm Society (HRS), Asia Pacific Heart Rhythm Society (APHRS), Cardiac Arrhythmia Society of Southern Africa (CASSA), and Latin America Heart Rhythm Society (LAHRS). *Europace* 2019;doi:10.1093/europace/euz046. 10.1093/europace/euz046 30882141

[8] Kirchhof P, Camm AJ, Goette A, Brandes A, Eckardt L, Elvan A, Fetsch T, van Gelder IC, Haase D, Haegeli LM, Hamann F, Heidbuchel H, Hindricks G, Kautzner J, Kuck KH, Mont L, Ng GA, Rekosz J, Schoen N, Schotten U, Suling A, Taggeselle J, Themistoclakis S, Vettorazzi E, Vardas P, Wegscheider K, Willems S, Crijns H, Breithardt G; EAST-AFNET 4 Trial Investigators. Early rhythm-control therapy in patients with atrial fibrillation. N Engl J Med 2020;383:1305–1316. 32865375 10.1056/NEJMoa2019422

[9] Hohnloser SH, Crijns HJ, van Eickels M, Gaudin C, Page RL, Torp-Pedersen C, Connolly SJ. Effect of dronedarone on cardiovascular events in atrial fibrillation. N Engl J Med 2009;360:668–678. 19213680 10.1056/NEJMoa0803778

[10] Kirchhof P, Breithardt G, Camm AJ, Crijns HJ, Kuck KH, Vardas P, Wegscheider K. Improving outcomes in patients with atrial fibrillation: rationale and design of the Early treatment of Atrial fibrillation for Stroke prevention Trial. Am Heart J 2013;166:442–448. 24016492 10.1016/j.ahj.2013.05.015

[11] January CT, Wann LS, Calkins H, Chen LY, Cigarroa JE, Cleveland JC Jr., Ellinor PT, Ezekowitz MD, Field ME, Furie KL, Heidenreich PA, Murray KT, Shea JB, Tracy CM, Yancy CW. 2019 AHA/ACC/HRS Focused Update of the 2014 AHA/ACC/HRS Guideline for the management of patients with atrial fibrillation: a report of the American College of Cardiology/American Heart Association Task Force on Clinical Practice Guidelines and the Heart Rhythm Society in Collaboration With the Society of Thoracic Surgeons. Circulation 2019;140:e125–e151. 30686041 10.1161/CIR.0000000000000665

[12] Wyse DG, Waldo AL, DiMarco JP, Domanski MJ, Rosenberg Y, Schron EB, Kellen JC, Greene HL, Mickel MC, Dalquist JE, Corley SD; Atrial Fibrillation Follow-up Investigation of Rhythm Management (AFFIRM) Investigators. A comparison of rate control and rhythm control in patients with atrial fibrillation. N Engl J Med 2002 Dec 5;347:1825–1833. 33. doi: 10.1056/NEJMoa021328. PMID: 12466506. 12466506

[13] Van Gelder IC, Hagens VE, Bosker HA, Kingma JH, Kamp O, Kingma T, Said SA, Darmanata JI, Timmermans AJM, Tijssen JGP, Crijns HJGM; Rate Control versus Electrical Cardioversion for Persistent Atrial Fibrillation Study Group. A comparison of rate control and rhythm control in patients with recurrent persistent atrial fibrillation. N Engl J Med 2002;347:1834–1840. 12466507 10.1056/NEJMoa021375

[14] Roy D, Talajic M, Nattel S, Wyse DG, Dorian P, Lee KL, Bourassa MG, Arnold JM, Buxton AE, Camm AJ, Connolly SJ, Dubuc M, Ducharme A, Guerra PG, Hohnloser SH, Lambert J, Le Heuzey JY, O'Hara G, Pedersen OD, Rouleau JL, Singh BN, Stevenson LW, Stevenson WG, Thibault B, Waldo AL; Atrial Fibrillation and Congestive Heart Failure Investigators. Rhythm control versus rate control for atrial fibrillation and heart failure. N Engl J Med 2008;358:2667–2677. 18565859 10.1056/NEJMoa0708789

[15] Yang E, Tang O, Metkus T, Berger RD, Spragg DD, Calkins HG, Marine JE. The role of timing in treatment of atrial fibrillation: an AFFIRM substudy. Heart Rhythm 2021;18:674–681. 33383228 10.1016/j.hrthm.2020.12.025

[16] Marrouche NF, Brachmann J, Andresen D, Siebels J, Boersma L, Jordaens L, Merkely B, Pokushalov E, Sanders P, Proff J, Schunkert H, Christ H, Vogt J, Bansch D, Investigators C-A; CASTLE-AF Investigators. Catheter ablation for atrial fibrillation with heart failure. N Engl J Med 2018;378:417–427. 29385358 10.1056/NEJMoa1707855

[17] Packer DL, Mark DB, Robb RA, Monahan KH, Bahnson TD, Poole JE, Noseworthy PA, Rosenberg YD, Jeffries N, Mitchell LB, Flaker GC, Pokushalov E, Romanov A, Bunch TJ, Noelker G, Ardashev A, Revishvili A, Wilber DJ, Cappato R, Kuck KH, Hindricks G, Davies DW, Kowey PR, Naccarelli GV, Reiffel JA, Piccini JP, Silverstein AP, Al-Khalidi HR, Lee KL; CABANA Investigators. Effect of catheter ablation vs antiarrhythmic drug therapy on mortality, stroke, bleeding, and cardiac arrest among patients with atrial fibrillation: the CABANA randomized clinical trial. JAMA 2019;321:1261–1274. 30874766 10.1001/jama.2019.0693 PMC6450284

[18] Cosedis Nielsen J, Johannessen A, Raatikainen P, Hindricks G, Walfridsson H, Kongstad O, Pehrson S, Englund A, Hartikainen J, Mortensen LS, Hansen PS. Radiofrequency ablation as initial therapy in paroxysmal atrial fibrillation. N Engl J Med 2012;367:1587–1595. 23094720 10.1056/NEJMoa1113566

[19] Wilber DJ, Pappone C, Neuzil P, De Paola A, Marchlinski F, Natale A, Macle L, Daoud EG, Calkins H, Hall B, Reddy V, Augello G, Reynolds MR, Vinekar C, Liu CY, Berry SM, Berry DA; ThermoCool AF Trial Investigators. Comparison of antiarrhythmic drug therapy and radiofrequency catheter ablation in patients with paroxysmal atrial fibrillation: a randomized controlled trial. JAMA 2010;303:333–340. 20103757 10.1001/jama.2009.2029

[20] Andrade JG, Wells GA, Deyell MW, Bennett M, Essebag V, Champagne J, Roux JF, Yung D, Skanes A, Khaykin Y, Morillo C, Jolly U, Novak P, Lockwood E, Amit G, Angaran P, Sapp J, Wardell S, Lauck S, Macle L, Verma A; EARLY-AF Investigators. Cryoablation or drug therapy for initial treatment of atrial fibrillation. N Engl J Med 2021;384:305–315. 33197159 10.1056/NEJMoa2029980

[21] Wazni OM, Dandamudi G, Sood N, Hoyt R, Tyler J, Durrani S, Niebauer M, Makati K, Halperin B, Gauri A, Morales G, Shao M, Cerkvenik J, Kaplon RE, Nissen SE; STOP AF First Trial Investigators. Cryoballoon ablation as initial therapy for atrial fibrillation. N Engl J Med 2021;384:316–324. 33197158 10.1056/NEJMoa2029554

[22] Metzner A, Suling A, Brandes A, Breithardt G, Camm AJ, Crijns HJGM, Eckardt L, Elvan A, Goette A, Haegeli LM, Heidbuchel H, Kautzner J, Kuck KH, Mont L, Ng GA, Szumowski L, Themistoclakis S, van Gelder IC, Vardas P, Wegscheider K, Willems S, Kirchhof P. Components of AF management and early rhythm control in patients with atrial fibrillation: a detailed analysis of the EAST-AFNET 4 dataset. *Europace*, in press.

[23] Rienstra M, Vermond RA, Crijns HJ, Tijssen JG, Van Gelder IC RACE Investigators. Asymptomatic persistent atrial fibrillation and outcome: results of the RACE study. Heart Rhythm 2014;11:939–945. 24632222 10.1016/j.hrthm.2014.03.016

[24] Flaker GC, Belew K, Beckman K, Vidaillet H, Kron J, Safford R, Mickel M, Barrell P; AFFIRM Investigators. Asymptomatic atrial fibrillation: demographic features and prognostic information from the Atrial Fibrillation Follow-up Investigation of Rhythm Management (AFFIRM) study. Am Heart J 2005;149:657–663. 15990749 10.1016/j.ahj.2004.06.032

[25] Wazni OM, Marrouche NF, Martin DO, Verma A, Bhargava M, Saliba W, Bash D, Schweikert R, Brachmann J, Gunther J, Gutleben K, Pisano E, Potenza D, Fanelli R, Raviele A, Themistoclakis S, Rossillo A, Bonso A, Natale A. Radiofrequency ablation vs antiarrhythmic drugs as first-line treatment of symptomatic atrial fibrillation: a randomized trial. JAMA 2005;293:2634–2640. 15928285 10.1001/jama.293.21.2634

[26] Mark DB, Anstrom KJ, Sheng S, Piccini JP, Baloch KN, Monahan KH, Daniels MR, Bahnson TD, Poole JE, Rosenberg Y, Lee KL, Packer DL; CABANA Investigators. Effect of catheter ablation vs medical therapy on quality of life among patients with atrial fibrillation: the CABANA randomized clinical trial. JAMA 2019;321:1275–1285. 30874716 10.1001/jama.2019.0692 PMC6450275

[27] Wynn GJ, Das M, Bonnett LJ, Gupta D. Quality-of-life benefits of catheter ablation of persistent atrial fibrillation: a reanalysis of data from the SARA study. Europace 2015;17:222–224. 25028177 10.1093/europace/euu154

