# Supplementary material for: Systematic, early rhythm control strategy for atrial fibrillation in patients with or without symptoms: the EAST-AFNET 4 trial
Source: Eur Heart J. 2021 Aug 27;43(12):1219–30. doi: 10.1093/eurheartj/ehab593 (PMC8934687; doi:10.1093/eurheartj/ehab593)
Supplement: ehab593_Supplementary_Data [file ehab593_supplementary_data.pdf]

Systematic, early rhythm control therapy equally improves outcomes in asymptomatic and symptomatic patients with atrial fibrillation: the EAST-AFNET 4 Trial

DATA SUPPLEMENT

Table 1. Number of patients split by randomized group and EHRA score at baseline

	**Overall**, N = 2,789[1]	Early rhythm control, N = 1395[1]	Usual care, N = 1394[1]	**p-value**[2]
EHRA at baseline				0.455
I	801 (30%)	395 (30%)	406 (31%)	
II	1,358 (52%)	666 (51%)	692 (52%)	
III	447 (17%)	230 (18%)	217 (16%)	
IV	27 (1.0%)	14 (1.1%)	13 (1.0%)	
Unknown	156	90	66	

[1] n (%)

[2] Comparison between the different treatment groups were performed using mixed ordinal regression with site as random effect.

Table 2. Demographic and clinical characteristics of the patients at Baseline, split into asyomptomatic and symptomatic patients

	Asymptomatic AF		Symptomatic AF		
	Early rhythm control	Usual care	Early rhythm control	Usual care	p (asymp vs. symp)
n	395	406	910	922	
	**Characteristics**				
Age (mean (SD))	71.0 (8.0)	71.5 (7.9)	70.0 (8.5)	70.0 (8.3)	0.002
Women	138 (34.9)	162 (39.9)	470 (51.6)	453 (49.1)	<0.001
Body Mass Index (mean (SD))	29.0 (5.1)	29.5 (5.6)	29.2 (5.5)	29.3 (5.3)	0.689
AF type					<0.001*
First episode	195/395 (49.4)	195/406 (48.0)	322/910 (35.4)	317/922 (34.4)	
Paroxysmal	103/395 (26.1)	98/406 (24.1)	347/910 (38.1)	353/922 (38.3)	
Persistent or long-standing persistent	97/395 (24.6)	113/406 (27.8)	241/910 (26.5)	252/922 (27.3)	
Median days since first diagnosis of AF(IQR)	21.0 (4.0,79.0)	23.5 (4.0, 96.0)	40.0 (7.0, 121.8)	40.0 (6.0, 118.8)	0.002
Previous pharmacological or electrical cardioversion	133/385 (34.5)	143/403 (35.5)	388/894 (43.4)	382/920 (41.5)	<0.001
Ventricular rate in AF (average of 10 intervals)	92.2 (26.2)	94.8 (27.7)	95.7 (24.9)	100.5 (28.2)	0.019
	**Concomitant cardiovascular conditions**				
Previous stroke or transient ischemic attack	61/395 (15.4)	55/406 (13.5)	100/910 (11.0)	87/922 (9.4)	0.003
At least mild cognitive impairment	183/384 (47.7)	200/393 (50.9)	368/864 (42.6)	359/883 (40.7)	0.02
MoCA Score					0.008
None	201 / 384 (52)	193 / 393 (49)	496 / 864 (57)	524 / 883 (59)	
Mild	170 / 384 (44)	173 / 393 (44)	334 / 864 (39)	339 / 883 (38)	
Moderate	13 / 384 (3.4)	25 / 393 (6.4)	30 / 864 (3.5)	18 / 883 (2.0)	
Severe	0 / 384 (0)	2 / 393 (0.5)	4 / 864 (0.5)	2 / 883 (0.2)	
Arterial hypertension	338/395 (85.6)	355/406 (87.4)	810/910 (89.0)	803/922 (87.1)	0.348
Systolic blood pressure (mean (SD))	136.7 (19.6)	138.3 (18.9)	136.6 (19.5)	137.0 (19.4)	0.602
Diastolic blood pressure (mean (SD))	80.5 (12.7)	81.4 (12.2)	81.0 (11.8)	81.2 (11.9)	0.739
Stable heart failure	81/395 (20.5)	88/406 (21.7)	278/910 (30.5)	291/922 (31.6)	<0.001

	Asymptomatic AF		Symptomatic AF		
	Early rhythm control	Usual care	Early rhythm control	Usual care	p (asymp vs. symp)
CHA2DS2-VASc score (mean (SD))	3.4 (1.3)	3.4 (1.4)	3.3 (1.3)	3.3 (1.3)	0.558
Chronic kidney disease of MDRF stage 3 or 4	47/395 (11.9)	57/406 (14.0)	115/910 (12.6)	118/922 (12.8)	0.99
**Medication at discharge**					
Oral anticoagulation with NOAC or VKA	351/395 (88.9)	372/406 (91.6)	838/910 (92.1)	817/922 (88.6)	0.095
Digoxin or digitoxin	9/395 (2.3)	15/406 (3.7)	35/910 (3.8)	70/922 (7.6)	0.001
Beta blockers	282/395 (71.4)	342/406 (84.2)	711/910 (78.1)	795/922 (86.2)	0.004
ACE inhibitors or angiotensin II receptor blocker	286/395 (72.4)	294/406 (72.4)	616/910 (67.7)	642/922 (69.6)	0.201
Mineralocorticoid receptor antagonist	23/395 (5.8)	30/406 (7.4)	60/910 (6.6)	57/922 (6.2)	0.612
Diuretic	156/395 (39.5)	166/406 (40.9)	376/910 (41.3)	369/922 (40.0)	0.581
Statin	210/395 (53.2)	189/406 (46.6)	384/910 (42.2)	356/922 (38.6)	<0.001
Platelet inhibitor	79/395 (20.0)	67/406 (16.5)	140/910 (15.4)	151/922 (16.4)	0.153
**AF Therapy**					
Planned therapy for rhythm control at baseline					0.341*
AAD	354/395 (89.6)	15/406 (3.7)	787/910 (86.5)	37/922 (4.0)	
Ablation	24/395 (6.1)	1/406 (0.2)	78/910 (8.6)	1/922 (0.1)	
None	17/395 (4.3)	390/406 (96.1)	45/910 (4.9)	884/922 (95.9)	

MoCA Score Categories: None : >= 26; Mild: 18-25; Moderate: 10-17; Severe: < 10

Table 3: Outcomes split into symptomatic and asymptomatic patients and by randomized group.

	**Asymptomatic AF**			**Symptomatic AF**			
Outcome	Early rhythm control	Usual care	Treatment effect	Early rhythm control	Usual care	Treatment effect	p-Value interaction
First primary outcome	79/1888 (4.2)	97/1774 (5.5)	0.76 (0.57, 1.03)	158/4155 (3.8)	205/4307 (4.8)	0.79 (0.64, 0.98)	0.848
**Components of the first primary outcome**							
Death from cardiovascular causes	19/2049 (0.9)	22/1977 (1.1)	0.82 (0.44, 1.51)	45/4485 (1)	66/4742 (1.4)	0.72 (0.49, 1.05)	0.723
Stroke	19/2005 (0.9)	25/1923 (1.3)	0.73 (0.4, 1.33)	20/4429 (0.5)	34/4670 (0.7)	0.62 (0.36, 1.08)	0.701
Hospitalization with worsening of heart failure	39/1961 (2)	54/1845 (2.9)	0.67 (0.44, 1.01)	93/4289 (2.2)	108/4455 (2.4)	0.89 (0.67, 1.18)	0.255
Hospitalization with acute coronary syndrome	22/2004 (1.1)	18/1929 (0.9)	1.19 (0.64, 2.22)	28/4389 (0.6)	44/4619 (1)	0.67 (0.42, 1.08)	0.151
Secondary primary outcome: Nights spent in hospital/yr	5.5 (17.9)	6.1 (19.2)	0.91 (0.72, 1.16)	6.3 (24.4)	4.7 (14.2)	1.2 (1.03, 1.41)	0.061
**Key secondary outcomes at 2 years follow-up**							
Change in left ventricular ejection fraction	0.6 (10.4)	-0.5 (9.8)	0.18 (-1, 1.36)	2 (9.7)	1.2 (9.8)	0.25 (-0.57, 1.07)	0.919
Change in EQ-5D score	1.6 (16.7)	-1.2 (17.2)	1.53 (-1.74, 4.8)	1.3 (17.6)	1.5 (16.8)	0.89 (-1.27, 3.05)	0.755
Change in SF-12 Mental Score	1 (9.7)	1.7 (9.5)	-0.83 (-2.34, 0.67)	0.8 (10.9)	1.7 (10.2)	-1.36 (-2.33, -0.38)	0.556
Change in SF-12 Physical Score	-0.4 (8.3)	-1.2 (8.4)	0.84 (-0.57, 2.24)	0.7 (8.5)	0.7 (8)	0.11 (-0.73, 0.95)	0.385
Change in MoCA score	0.2 (3.4)	0.1 (3)	0.05 (-0.42, 0.53)	0 (3.2)	0.1 (3.3)	-0.22 (-0.53, 0.09)	0.354
Sinus rhythm	255/323 (78.9)	170/325 (52.3)	3.66 (2.56, 5.24)	616/729 (84.5)	484/759 (63.8)	2.93 (2.26, 3.79)	0.317

Note: Continuous variables were presented as the mean and standard deviation. See main paper for detailed description of the outcomes.
